# Artificial Olfactory Biohybrid System: An Evolving Sense of Smell

**DOI:** 10.1002/advs.202204726

**Published:** 2022-12-18

**Authors:** Chuanting Qin, Yi Wang, Jiawang Hu, Ting Wang, Dong Liu, Jian Dong, Yuan Lu

**Affiliations:** ^1^ Key Laboratory of Industrial Biocatalysis Ministry of Education Department of Chemical Engineering Tsinghua University Beijing 100084 China; ^2^ Tianjin Industrial Microbiology Key Laboratory College of Biotechnology Tianjin University of Science and Technology Tianjin 300457 China

**Keywords:** artificial olfactory system, bioelectronic nose, biomimicry, biosensors, volatile organic compounds

## Abstract

The olfactory system can detect and recognize tens of thousands of volatile organic compounds (VOCs) at low concentrations in complex environments. Bioelectronic nose (B‐EN), which mimics olfactory systems, is becoming an emerging sensing technology for identifying VOCs with sensitivity and specificity. B‐ENs integrate electronic sensors with bioreceptors and pattern recognition technologies to enable medical diagnosis, public security, environmental monitoring, and food safety. However, there is currently no commercially available B‐EN on the market. Apart from the high selectivity and sensitivity necessary for volatile organic compound analysis, commercial B‐ENs must overcome issues impacting sensor operation and other problems associated with odor localization. The emergence of nanotechnology has provided a novel research concept for addressing these problems. In this work, the structure and operational mechanisms of biomimetic olfactory systems are discussed, with an emphasis on the development and immobilization of materials. Various biosensor applications and current developments are reviewed. Challenges and opportunities for fulfilling the potential of artificial olfactory biohybrid systems in fundamental and practical research are investigated in greater depth.

## Graphical abstract

Benefiting from the gradual decipherment of olfaction, artificial olfactory biosensor systems are being developed for applications in industry, security, food, and medicine. Bioinspired receptors and diverse transducers are designed and evolved to improve sensing traces of odorants. Cutting‐edge technologies are integrated to overcome the biosensor challenges of sensitivity, selectivity, reproducibility, stability, and lifetime.

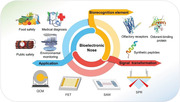

## 1 Introduction

In natural environments, animals typically encounter odors in a complex mixture of distinct components. The environment is rich in odorants emitted from various natural and unnatural sources (plants, bacteria, industrial activities, and other human activities).^[^ ^1^ ^]^ Odors are mainly composed of hydrophobic volatile organic compounds (VOCs) with molecular weights of less than 300 Da. One of the most prominent characteristics of olfactory perception is its triggering effect on the emotional and affective experience.^[^ ^2^ ^]^ Common examples may be the perfume of a beloved one immediately activating past affective memories or an unpleasant smell of foods that had made us feel sick, automatically triggering an aversive sensation.^[^ ^3^ ^]^ Industrial production and chemical plants are often the main sources of gaseous emissions. While these facilities may not pollute ambient air at concentrations higher than the limit for monitoring chemicals, they may produce mixtures of other compounds that cause odor pollution. Long‐term exposure to mixtures of volatile compounds (e.g., aromatic hydrocarbons, organic and inorganic sulfides, and nitrogen and halides) may represent a risk for different diseases, including asthma, atopic dermatitis, and neurological damage.^[^ ^4^ ^]^ Furthermore, identification of the markers in exhaled air has the potential for clinical application in many diseases, for instance, lungs, digestive system, and oncological and systemic diseases.^[^ ^5^ ^]^ Nowadays, the analysis of volatile organic compound (VOC) is of great interest to a diverse variety of fields, such as environmental monitoring, public safety and security, the food and beverage industry, the cosmetics and perfume industry, medical diagnostics and health monitoring, etc.

The traditional analytical methods, such as gas chromatography coupled to mass spectroscopy, are very accurate, reliable, and able to identify different substances in a sample, but these instruments are costly, complex, and bulky.^[^ ^6^ ^]^ Therefore, significant efforts have been devoted to mimicking natural olfactory systems to achieve high selectivity and sensitivity.

Although electronic nose (EN) has been applied to detect and discriminate different VOCs sensitively and rapidly,^[^ ^7^ ^]^ ENs are not per se feasible to measure odor qualities. The seeming similarity between the biological functional structure of the sense of smell and the construction of ENs falls short.^[^ ^8^ ^]^ On the contrary, ENs are chemical measurement systems that measure the chemical properties of sample gases, not odor properties. In addition, EN devices still present hardware and software challenges. Sensor types such as metal oxide semiconductor,^[^ ^7b^ ^]^ quartz crystal microbalance (QCM),^[^ ^9^ ^]^ conducting polymer,^[^ ^7c^ ^]^ colorimetric,^[^ ^10^ ^]^ and surface acoustic wave (SAW)^[^ ^11^ ^]^ are frequently used in EN devices. Unfortunately, these sensors are sensitive to temperature and humidity changes, which indeed are a serious drawback of ENs, especially in the food and beverage industries. As ambient humidity increases, the sensitivity of the sensor decreases, which may lead to unreliable responses from the EN, and odor samples with the same characteristics may be classified as different gases.^[^ ^12^ ^]^ Sensor drift, defined as the small changes in the output independent of the measured feature, is another issue in EN technology. Because of the short‐ and long‐term drift of the sensor, the pattern recognition ability is generally degraded.^[^ ^13^ ^]^ Drift can be offset to some extent, but usually through time‐consuming calibration or recalibration. Such as metal‐oxide gas sensors require regular recalibration, which leads to time waste and high economic costs.^[^ ^14^ ^]^ New developments in biotechnology, such as gene editing techniques, make it possible to develop odor sensors that can identify gases and VOCs in low concentrations with high selectivity by specifically modifying the biological receptor components.^[^ ^15^ ^]^ The gene editing techniques have provided a brilliant opportunity for precise intracellular gene manipulation, which can not only be used to induce mutations, corrections, or deletions but can also introduce foreign genes at specific sites.^[^ ^16^ ^]^ Recent research has shown that therapeutic gene editing techniques can restore olfactory cilia morphology and function in the olfactory sensory neuron (OSN), and further reconstruct odor‐directed behavior in animals.^[^ ^17^ ^]^ Recent research has shown that therapeutic gene editing techniques can restore olfactory cilia morphology and function in OSN, and further reconstruct odor‐directed behavior in animals.^[^ ^17^ ^]^ Currently, different investigations are carried out, including the digitalization of smell sensations and emotions accompanying particular odors and tastes. Some stages can be accomplished with the bioelectronic nose (B‐EN), which mimics the principle of operation of the human sense of smell in the most precise way due to the utilization of the olfactory receptors (ORs) as one of the measurement elements.^[^ ^18^ ^]^

**Figure 1:**
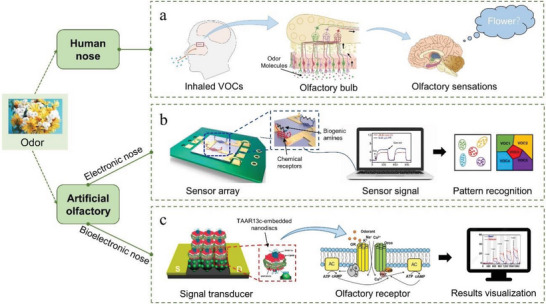
The similarities and differences between the EN, B‐EN, and human nose structures. a) The olfactory mechanism of humans. The initial events of olfactory perception occur in the olfactory epithelium (OE). Olfactory transduction begins with the activation of odor receptors located on the cilia of OSNs, which constitute the OE. Subsequently transmits the odor information to the olfactory bulb, which integrates the odor information and transmits it to the olfactory center, thus triggering the sense of smell. b) The mechanism of electronics nose. EN acquires VOC through an air pump and then transmits the VOC to the sensor platform, where the chemical material on the sensor recognizes the odor and then transmits the signal to a computer for processing. Reproduced with permission.^[^ ^24^ ^]^ Copyright 2022, Elsevier. c) The mechanism of bioelectronics nose. Same as EN, B‐EN also has to acquire VOC through air pump, but the sensor platform is composed of biological components that mimic human olfaction and bind specifically to the odor, generating a signal that is amplified and converted, and then the final result is visualized through a computer. Reproduced with permission.^[^ ^25^ ^]^ Copyright 2019, American Chemical Society

A principal assumption in the design of B‐EN is applying olfactory receptor (OR) as the active element of the sensor for VOC analysis with high sensitivity and specificity.^[^ ^19^ ^]^ The biosensor used in this type of nose is an analytical device for detecting analytes that combines biological components with a physicochemical detector.^[^ ^20^ ^]^ Compared with traditional analysis methods, biosensing technology has irreplaceable advantages. First, biosensors can interact with biological macromolecules in real‐time and analyze the changes occurring at any moment in the process. Second, the whole process only takes 5–15 min, and many samples can be measured in a short time.^[^ ^21^ ^]^ Analysis shows that in all application fields, the biosensor is better than chemical sensors to meet all standards in the field of application, including high specificity, high selectivity, high precision, and high sensitivity.^[^ ^22^ ^]^ So biological sensor technology in all application areas usually has a high application potential, especially in the aspect of health and safety.^[^ ^23^ ^]^ **Figure** **1** shows the similarities and differences between the EN, B‐EN, and human nose structures.

In recent years, B‐EN has been combined with a variety of sensing technologies and has been greatly developed. Though it highly depends on the function of ORs, it can realize the detection of most molecules in the environment. By combining biotechnology, nanotechnology, and microsystem technology, the sensitivity, specificity, and stability of B‐EN can be greatly improved, showing strong sensing ability. The B‐EN is not only widely used in traditional environmental monitoring,^[^ ^26^ ^]^ but also provides new ideas for physiological health monitoring, drug screening and development, explosives and narcotics detection, etc.^[^ ^27^ ^]^ For example, B‐ENs provide olfactory benefits to patients with anosmia and hyposmia, and will also contribute to various industries related to food, beverage, and flavor by providing objective olfactory information.^[^ ^28^ ^]^ The development of a composite B‐EN provides a new idea for realizing odor standardization. It can use pattern recognition technology to provide complex odor information and even can reproduce odor through an integrated olfactory display system.^[^ ^29^ ^]^ This review highlights information about the fundamentals of olfactory‐inspired biomaterials for application in odor biosensors and discusses future challenges and prospects for artificial olfactory biohybrid systems.

## 2 Development of Bioelectronic Nose

### 2.1 Design of Bioelectronic Nose

**Figure 2:**
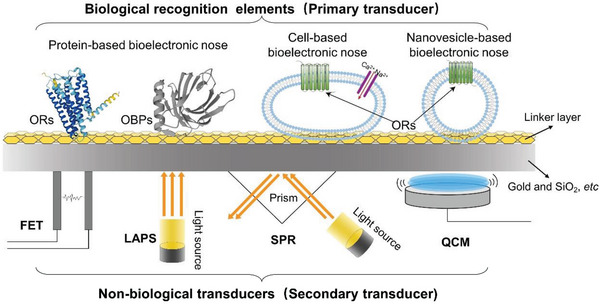
Composition of a B‐EN. The primary transducer contains ORs, odorant‐binding proteins (OBPs), cells, and nanovesicles. Secondary transducers include field‐effect transistors (FETs), light addressable potentiometric sensor (LAPS), surface plasmon resonance (SPR), QCM, etc.

The B‐EN directly utilizes the interaction between different targets and biomimetic materials to realize the bionic design in vitro through electrical signal processing.^[^ ^30^ ^]^ The B‐EN is mainly composed of a biological detection unit and a secondary sensor signal conversion platform, as shown in **Figure** **2**.

Biological or biomimetic receptor components, such as whole animals,^[^ ^31^ ^]^ insect tentacles,^[^ ^32^ ^]^ ORs,^[^ ^33^ ^]^ odorant‐binding protein (OBP),^[^ ^34^ ^]^ peptides,^[^ ^35^ ^]^ olfactory cells and tissues,^[^ ^36^ ^]^ molecularly imprinted polymers (MIPs),^[^ ^37^ ^]^ as the element of the biosensors, allow a significant improvement of selectivity and specificity with simultaneous reduction of the problems associated with cross‐reactivity and complex sample matrix. The secondary transducers are non‐biological devices, which are used to convert and amplify biological signals. Surface plasmon resonance (SPR),^[^ ^38^ ^]^ field‐effect transistor (FET),^[^ ^39^ ^]^ light addressable potentiometric sensor (LAPS),^[^ ^40^ ^]^ QCM, SAW, electrochemical impedance spectroscopy (EIS), and other devices have been developed to be used as non‐biological sensors. Signal generation occurs as a result of contact between the odorous substance and the receptor, where adenyl cyclase (AC) is activated via a membrane G protein. Recognition elements made of olfactory biomaterials such as ORs are directly connected to sensors for odor recognition and convert the biological signal into an electrical or optical signal.^[^ ^18b^ ^]^ The biosignals acquired from ORs can be classified into three types: conformational changes in ORs, subunit alpha dissociation in activated G proteins, and ion influx caused by signal transduction in the cell.^[^ ^41^ ^]^ Non‐biosensors then detect these changes, which are then translated into changes in the resonant frequency, resonance angle, or electricity of the sensor. The most important point for the performance of B‐ENs is the coupling between the biometric element and the sensor.^[^ ^42^ ^]^ The commonly used immobilization methods are physical adsorption and chemical covalent binding. Physical adsorption is simpler and easier to perform than covalent binding, but the stability is relatively poor. In addition, covalent binding may induce conformational changes in protein structure and active sites, so this hinders their application to some extent. Therefore, the best immobilization method should be selected according to the characteristics of the biomimetic element to develop a B‐EN with good performance.^[^ ^43^ ^]^ Since the concept of B‐EN was proposed, various subject groups around the world have started to study B‐ENs, as shown in **Table** **1**. The Park group's research is more extensive and contains various biomimetic components as well as sensors and attempts to combine microfluidic systems with EN. Wang and colleagues have developed a novel in vivo B‐EN. Jadranka's group focuses on biosensors based on insect receptor proteins. In addition, the sensor group initiated by Corrado Di Natale has made great contributions to various types of biosensors. B‐ENs are booming, and there have been many impressive advances in the field, which will also have the potential to attract more researchers to join the ranks of biosensing and develop high‐quality B‐ENs using nanomaterials.

**Table 1:** Research on B‐ENs by various groups

Groups	Research content
Tai Hyun Prak	FET‐type B‐EN^[^ ^44^ ^]^
	Application of nanomaterials in B‐EN^[^ ^45^ ^]^
	Microfluidic system combined with B‐EN^[^ ^46^ ^]^
Tomasz Wasilewski	Peptide‐based B‐EN^[^ ^26^, ^47^ ^]^
Ping Wang	B‐EN is based on cells and OSNs^[^ ^48^ ^]^
	Research on in vivo B‐EN^[^ ^49^ ^]^
Jadranka Travas‐Sejdic	Insect odor receptor‐based biosensor^[^ ^32^, ^39^, ^50^ ^]^
Yanxia Hou	SPR‐type B‐EN^[^ ^38^, ^51^ ^]^
Corrado Di Natale	Various types of biosensors^[^ ^52^ ^]^

The B‐EN can accurately distinguish the target molecules in the mixture with good selectivity. A peptide receptor‐based bioelectronic nose (PRBN) designed by Lim et al. can not only detect trimethylamine (TMA) from spoiled seafood but also can be able to distinguish spoiled seafood from other types of spoiled food samples and fresh seafood samples.^[^ ^44a^ ^]^ In addition, B‐ENs with nanomaterials are capable of detecting gaseous odors at concentrations as low as 0.02 parts per trillion (ppt),^[^ ^45a^ ^]^ which is similar to the human nose. However, B‐ENs still face some challenges based on olfactory biological components. For example, since biomaterials are degradable, this will affect the lifetime of the B‐EN, and heterologous expression and bulk purification of proteins are not easy. Finally, stability and reproducibility are limitations of biosensors that also need to be addressed. One way to address the reproducibility of the sensor is to expose the OBPs to organic solvents such as ethanol and acetonitrile. Organic solvents not only denature proteins slightly and reversibly, thereby unfolding their binding pockets, but also dissolve VOCs and facilitate their evacuation from the binding pockets. Afterward, when the OBPs were returned to the phosphate buffer, their original structure and activity were restored. The results showed that the lifetime of the sensor was improved to about two months, which is remarkable compared to other B‐ENs in the literature.^[^ ^53^ ^]^ Besides, another way to solve this problem is to combine olfactory sensing materials with other non‐biosensing materials, such as nanomaterials and metal oxides. First, the silicon nanowire (SiNW) array was functionalized with Anopheles gambiae OBP (AgOBP), and then several cycles of response and recovery experimental results demonstrated the full recovery ability and reusability of the device.^[^ ^54^ ^]^

### 2.2 Diversification of Signal Transformation

The key point of biosensors is to convert biological signals generated by biological materials and odor molecules into detectable signals, so suitable detection instruments are also very major. With the development of complex detection technology and bionic technology, many mature sensors have been developed, which can be divided into three main signal categories, including an optical signal (e.g., SPR), an electrical signal (e.g., EIS, FET, and LAPS), and mass‐based measurement methods (e.g., QCM and SAW).

SPR can detect the binding interaction between biomaterials and VOCs on the sensor surface in real‐time and does not require biomarkers. The principle of SPR detection is based on total reflection, and when the SPR sensor surface is coated with biomaterials, the binding of VOCs to biomaterials affects the change in the angle of reflected light. The feasibility and effectiveness of such systems using biomolecules as materials to detect odor molecules in liquids have been demonstrated.^[^ ^50^, ^55^ ^]^ In recent years, two new detection methods, localized SPR (LSPR)^[^ ^56^ ^]^ and SPRi,^[^ ^57^ ^]^ have been proposed based on SPR.

FETs are often combined with nanotubes with high electrical conductivity and chemical stability for the detection of odorants, such as single‐walled carbon nanotube (SWCNT) and conducting polymer nanotube.^[^ ^58^ ^]^ In addition to nanotubes, graphene with a high specific surface area and good electrical conductivity is also used as a sensing material. Reduced graphene oxide (rGO) has undergone electrochemical reduction,^[^ ^59^ ^]^ which not only increases the conductivity of FET electrodes but also immobilizes proteins. Due to the excellent stability and sensitivity properties of nanomaterials and FETs, FETs using nanomaterials are already one of the most applied sensors today. EIS is one of the most sensitive electrochemical techniques, capable of delivering measurable signal changes resulting from small changes in biomarker concentration.^[^ ^60^ ^]^ Because of its surface charge detection capability, EIS is well suited for the development of biosensors and enables label‐free chemical sensing.^[^ ^45^, ^61^ ^]^ LAPS are semiconductor photoelectric effect‐based chemical sensors that are commonly used for the development of cell‐based biosensors,^[^ ^62^ ^]^ which can record potential changes outside the cell. In addition to LAPS, FETs, and microelectrode arrays (MEAs)^[^ ^63^ ^]^ can also be used to detect extracellular potentials. However, the reproducibility of such sensors is limited, so a lot of additional work is needed to improve reproducibility in the standard fabrication of sensors, such as improving the expression efficiency of OR in the OSN and modifications on the surface of LAPS.

Both QCM sensors and SAW sensors detect odor molecules by measuring the resonant frequency caused by the change in mass of the odorant after adsorption on the sensor surface. QCMs have been widely used in the development of various types of B‐ENs.^[^ ^50^, ^64^ ^]^ QCMs are more sensitive than SAWs and are capable of detecting concentrations up to the parts per million (ppm) level.^[^ ^65^ ^]^ To improve the sensitivity of SAW, a thin layer of diamond nanoparticles can also be coated on the surface of the sensor, detecting odors up to the parts per billion (ppb) level.^[^ ^66^ ^]^

**Figure 3:**
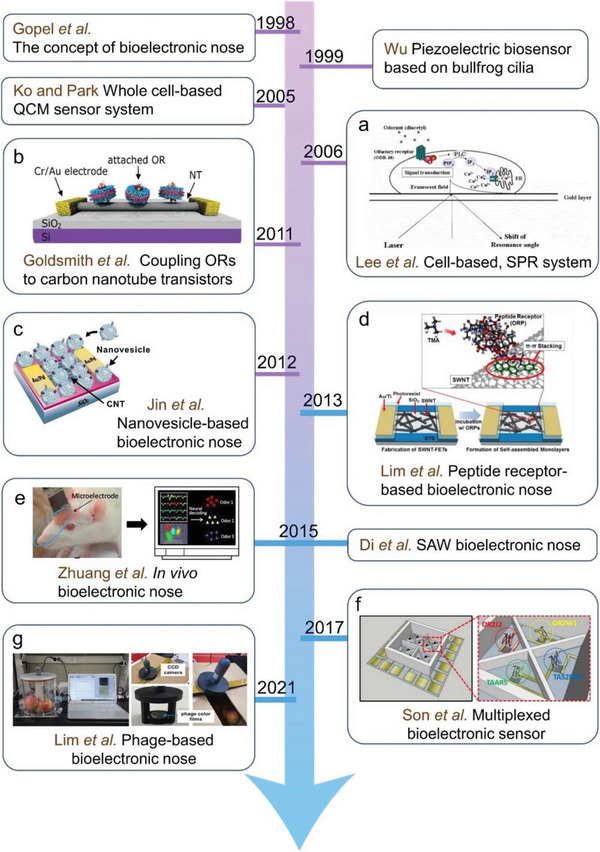
A brief history of the B‐ENs. a) Principle of cell‐based measurement of odorant molecules using SPR. Reproduced with permission.^[^ ^58b^ ^]^ Copyright 2006, Elsevier. b) Schematic of a carbon nanotube (CNT) transistor functionalized with mORs in nanodiscs (NDs). Reproduced with permission.^[^ ^67^ ^]^ Copyright 2011, American Chemical Society. c) Immobilization of a nanovesicle on CNT‐FET. Reproduced with permission.^[^ ^45d^ ^]^ Copyright 2012, Elsevier. d) PRBN for the detection of TMA. Reproduced with permission.^[^ ^44a^ ^]^ Copyright 2013, Elsevier. e) Schematic of in vivo B‐EN. Reproduced with permission.^[^ ^49b^ ^]^ Copyright 2015, Elsevier. f) Schematic diagram of an olfactory and taste receptor‐functionalized multichannel‐type CNT‐FET platform. Reproduced with permission.^[^ ^68^ ^]^ Copyright 2017, Elsevier. g) Photographs composed of a phage‐based B‐EN. Reproduced with permission.^[^ ^69^ ^]^ Copyright 2021, Elsevier.

After the concept of B‐EN was proposed by Goepl et al. in 1998, the development of B‐EN based on the QCM system using OR was attempted in 1999. Subsequently, researchers have developed B‐ENs based on various biomaterials and detection systems. In 2021 Lim et al. developed a new B‐EN composed of phages. A brief history of the B‐EN is shown in **Figure** **3**.

## 3 Biorecognition Element Design and Selection

For the development of B‐ENs using biomaterials, there is a need to obtain functional bioreceptors that maintain their unique chemical sensing capabilities and are suitable as recognition elements for biosensors. This section will examine and discuss various methods of production and immobilization of biorecognition receptors.

### 3.1 Olfactory Receptors

**Figure 4:**
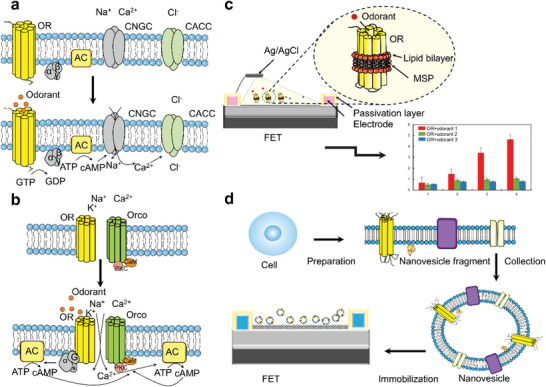
a) Signal transduction of odor responses. The OR catalyzes the release of the *G*_olf_ subunit, which in turn activates AC, leading to an increase in the cAMP levels that activate cyclic nucleotide‐gated channel, the calcium channel responsible for the depolarization, and the chloride channel that opens after the surge of Ca^2+^ into the cell. b) Illustrative depiction of the insect odorant receptor (OR)/Orco complex. Binding of odorants with OR triggers conformational changes in this complex and induces an influx of cations, which evoke depolarization of the receptor membrane potential. c) OR embedded ND, immobilized in the transducer. The ND consists of receptor, lipid, and membrane scaffold protein. The C‐terminus of the OR is anchored to the nanotube wall on the sensor surface via *π*–*π* interactions. d) Schematic diagram of olfactory signals generated by nanovesicles. The receptor protein‐containing cells were fragmented using artificial olfactory cells Cytochalasin B. Spherical olfactory nanovesicles with all components of the olfactory signal were collected, and the receptor protein‐containing nanovesicles were immobilized on SWCNT‐FET to collect the signals generated by the nanovesicles in response to specific odors.

ORs consist of proteins located in the cell membrane or cytoplasmic part of the cell whose purpose is to bind to odor molecules called ligands. Many large and small clusters of ORs are distributed throughout the genome. ORs are expressed in a highly specific manner and monogenic expression is the general rule, that is, one neuron, one receptor. Any particular OR gets activated by a handful of different compounds, not just by a single compound.^[^ ^70^ ^]^ About 1000 functional ORs are known to exist in vertebrates or insect animals.^[^ ^71^ ^]^ Currently, only 10% of ORs have been isolated and purified as recognition elements in sensors, and most of them are still in the experimental research stage. In vertebrates, ORs belong to the seven transmembranes G‐protein coupled receptor (GPCR),^[^ ^72^ ^]^ thus, odor signals are translated into electrical signals via a heterotrimeric G protein‐mediated second messenger pathway (**Figure** **4**a). Odorants are bound by odorant receptor proteins embedded in the cilia membrane. Odorant binding induces the G_*α*olf_ protein to release GDP, bind GTP, and dissociate from the *β* and *γ* subunits. The activated G_*α*olf_ protein forms a complex with AC, which converts ATP into cyclic AMP (cAMP). The accumulation of cAMP triggers the opening of cyclic nucleotide‐gated channels (CNGCs), which allow for an influx of Na^+^ and Ca^2+^ across the membrane. The influx of Ca^2+^ enables the opening of Ca^2+^‐activated chloride channels and the efflux of Cl across the membrane. In short, the coordinated movement of ions in response to odorant‐binding depolarizes the OSN membrane potential and initiates an axon potential that is transmitted down the axon of the OSN. This action potential is relayed to neurons in the olfactory bulb and processed within the central nervous system as odorant sensory information. Unlike vertebrates, insect ORs and their odorant receptor coreceptor (Orco) form a heterodimer or complex that acts as an odor‐gated nonselective cation channel volatile.^[^ ^73^ ^]^ The binding of the odorant to this complex triggers a conformational change in the complex and induces cation influx, which causes depolarization of the receptor membrane potential (Figure 4b). Since insect ORs act as odor‐gated ion channels, they can convert odor signals to electrical signals on their own, without needing additional signaling mechanisms, such as G proteins, enzymes, and ion channels.^[^ ^74^ ^]^ This simple olfactory transduction mechanism makes insect ORs a good candidate for building sensor elements. ORs as recognition elements for sensors have the following advantages. 1) The use of genetic engineering enables the insertion of tags in ORs for sensor recognition and immobilization. 2) ORs produce specific potential changes when they bind to odorant molecules. 3) Their binding can be detected by optoelectronic devices. The use of ORs as recognition elements for B‐EN sensors allows the use of biologically optimized molecular recognition systems for odorant substances.

In the development of OR‐based biosensors, the activity of functional ORs can directly influence the performance of biosensors. Extracting ORs from living olfactory mucosa tissues and sensory cells is the most direct and convenient method for utilizing them as sensing elements of biosensors. Although this production method can maintain the natural structure of OR and its function, it is difficult to obtain specific sensitive elements with the desired OR.^[^ ^75^ ^]^ With the development of genetic engineering and genomic analysis, many studies have artificially produced ORs for odor detection.^[^ ^69^, ^76^ ^]^ ORs can be expressed and purified by heterologous systems. Recombinant vectors are constructed by inserting genes of specific ORs, and many types of heterologous expression systems have been developed so far, including *Escherichia coli*, yeast, and mammalian cells, which allow for large‐scale production and efficient purification processes.^[^ ^77^ ^]^ The genetic tractability of *E. coli* allows a variety of expression plasmids to be used to tune protein expression levels. This can be particularly important with membrane proteins when saturation of the translocon can be a rate‐limiting step. The use of *E. coli* to express recombinant proteins has the advantages of low cost, high expression, and short time consumption. However, depending on the properties of the protein, most of the expressed proteins are localized in the non‐functional state of the inclusion bodies, which poses a great challenge for subsequent purification and immobilization. *Saccharomyces cerevisiae* strain is an alternative method of protein production, which has advantageous features suitable for heterologous eukaryotic protein overexpression. Yeast cells have been used as a functional expression system for membrane proteins such as ORs and ion channels. Pajot‐Augy et al. expressed rat OR, I7, and G*α* proteins in the yeast cells and induced cell growth according to the expression of a resistance gene induced by the interaction between I7 and heptanal under a selective medium lacking histidine.^[^ ^78^ ^]^ In addition to the low cost and high level of expression using the yeast system, it is also possible to express eukaryotic post‐translationally modified proteins. However, the yeast expression system also encounters the same drawbacks as the *E. coli* expression system. Mammalian cells have been generally used to express mammalian ORs because they can facilitate the glycosylation and proper folding of eukaryotic proteins. For example, the human OR (hOR1A1) is heterologously expressed in human embryonic kidney 293 cells (HEK293), and the expression of hOR1A1 is tenfold higher than in olfactory cells or tissue extracts.^[^ ^79^ ^]^ In addition to human embryonic cells, ORs are also expressed using insect cells (SF21 cells and COS‐7).^[^ ^80^ ^]^ In recent years, cell‐free protein expression systems have emerged, providing an alternative approach to address heterologous system expression.^[^ ^81^ ^]^ It takes only a few hours to complete the integrated expression process. This system simply mimics the natural cytoplasmic environment, so it does not require living cells, and therefore it avoids toxic effects known from traditional cell‐based expression.^[^ ^82^ ^]^ Due to the low yield of expressed OR proteins and high production costs, fewer studies are using cell‐free systems in the field of olfactory proteins. As a complementary method, chemical synthesis can also be used to produce peptides and proteins to develop olfactory biosensors. Most ORs require the help of olfactory‐specific chaperones to be correctly targeted to the surface of heterologous cells.^[^ ^70a^ ^]^ This is one of the reasons why the heterologous expression of ORs is technically difficult when compared with the expression of nonolfactory GPCRs and also the reason why the majority of ORs remain orphan, that is, have no known ligands. Whether the ectopic ORs require endogenous chaperones or specific accessory factors to be functionally expressed in nonolfactory cell types remain to be determined.

Functional coupling between the OR and the sensor materials is critical to the performance of biosensors. The ideal method of immobilizing ORs on the sensor surface requires specific binding, which can greatly improve the sensitivity, stability, and reproducibility of biosensors. ORs as biosensor recognition elements are typically located in lipid bilayers.^[^ ^83^ ^]^ ORs always maintain their natural membrane environment within the membrane, thus avoiding the risk of structural changes or activity loss that may occur during OR immobilization. ORs bind to the sensor surface indirectly through the interaction of their surrounding lipid bilayer with the substrate, rather than directly through the binding of their amino acid chains. This property ensures that the receptor binding site of the odorant is still allowed to be accessible and functional.^[^ ^58a^ ^]^ Currently, artificial lipid bilayers are commonly used to immobilize OR proteins. A recent study demonstrated the use of mosquito ORs sensitive to 1‐often‐3‐ol, integrated into bilayer lipid membranes (BLMs) in a chamber device equipped with electrodes. Current changes caused by specific OR responses to octanol were obtained when the sensor was exposed to odorant concentrations of 0.01–0.2 ppm.^[^ ^84^ ^]^ In addition to using artificial lipid bilayers to immobilize ORs, nanodisks (NDs) and nanovesicles can also immobilize OR on a suitable transducer (Figure 4c). It was shown that the immobilization of nanomaterials does not hinder the activity of the receptor.^[^ ^58a^ ^]^ Nanomaterials can consist of soluble self‐assembled synthetic phospholipid bilayers containing transmembrane proteins that provide a stable environment for integral membrane proteins such as OR.^[^ ^85^ ^]^ Conformational changes between ORs and odorants lead to changes in the current of the nanomaterials and amplify such changes, enhancing the performance of the sensors.^[^ ^44g^ ^]^ The NDs are mostly used for ORs from insects and are unsuitable for mammalian ORs with large sizes.^[^ ^45d^ ^]^ For such cases, researchers have proposed ORs embedded in nanovesicles (Figure 4d), which can generate signals similar to those produced by cells. In addition, nanovesicles have some properties of protein materials, such as mass production and easy preservation.^[^ ^41^ ^]^ Due to their small size, nanovesicles can be coupled to nanomaterials to functionalize their surfaces.^[^ ^86^ ^]^ Son et al. constructed SWCNT‐FETs biosensors for real‐time monitoring of water quality using nanovesicles of two human ORs (hOR51S1 and hOR3A4).^[^ ^44e^ ^]^ Whether embedded in an artificial lipid bilayer or an ND, ORs can be coupled to several different types of transducers.

Since mammalian ORs are GPCRs, and insect ORs are ligand‐gated ion channels, sensors based on different types of ORs have their advantages and disadvantages. For example, one advantage of using mammalian ORs is the ability to amplify signals through metabolic signaling pathways. Thus, it is possible to develop more sensitive odor‐sensing systems. However, it is difficult to fully reconstruct the complex signaling pathways starting from ORs in heterologous cells. On the other hand, one advantage of using insect ORs is the ability to rapidly induce ion channel in‐flow after interacting with odorants. Therefore, it is possible to develop odor‐sensing systems with a faster response time. Whether mammalian or insect ORs are used as sensor recognition elements, they are faced with the problems of complex OR structure, difficulties in production and purification, and short lifetime. OR‐based biosensors have been applied to detect explosives, medicine, food, and agriculture (**Table** **2**).

**Table 2:** OR‐based biosensors

Protein	System	VOCs	Immobilization technique	Transducer	Limit of detection	Application	Refs.
hOR1A2	*E. coli*	Geraniol	CNT	FET	1 fm	Fragrance development	[45f]
DmelOR10a	SE21 cell	Methyl salicylate, methyl hexanoate, 4‐ethylguaiacol	Gold sensor crystals	QCM	1 fm	Food screening	[50a]
ODR‐10	Cell‐free	Diacetyl	CNT	EIS	10 µm	Liver cancer	[82]
TAAR13	*E. coli*	Ethanolamine, CV, TMA	CNT	FET	10 pm	Autopsy	[45e]
AeaegyOR7	*E. coli*	1‐Octen‐3‐ol	BLM	SiNW	0.01 ppm	Breath analysis	[87]
DmOR22a	*E. coli*	Ethyl hexanoate	Gold sensor crystals	QCM	5.5 fm	Pest control	[88]
MOR256‐17	SE21 cell	2,4‐Dinitrotoluene	–	MEA	–	Explosive detection	[89]
OR7D4	*Saccharomyces cerevisiae*	2‐Octanone, acetophenone	Boron doped diamond	EIS	1 µm	Environmental testing	[90]

### 3.2 Odorant Binding Proteins

**Figure 5:**
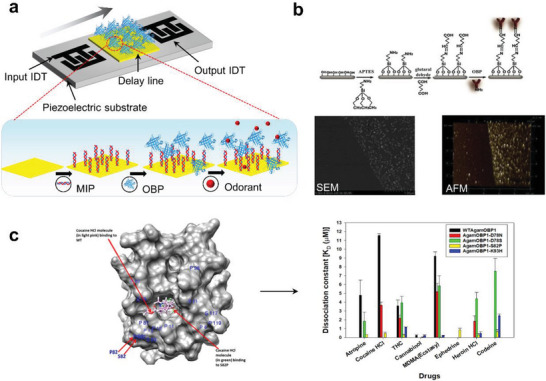
a) SAW biosensor configuration and surface functionalization method. b) Schematic illustration of SiNW surface modification process. Scanning electron microscopy and atomic force microscope image of the silicon surface with AgOBP modification, the step graph on the right shows the surface height in the white frame on the left. Reproduced with permission.^[^ ^54^ ^]^ Copyright 2020, American Chemical Society. c) Dissociation constants of WTAgamOBP1 and its mutant variants AgamOBP1‐D78N, AgamOBP1‐D78S, AgamOBP1‐S82P, AgamOBP1‐K93H toward the tested ligands atropine, cocaine–HCl, THC, cannabinol, MDMA, ephedrine, heroin–HCl, codeine. Reproduced under the terms of the CC BY license.^[^ ^55a^ ^]^ Copyright 2020, The Authors. Published by Springer Nature.

It was an interesting coincidence that OBPs were first identified at about the same time in the nasal cavity of mammals and the antennae of insects.^[^ ^91^ ^]^ OBPs are soluble proteins that act as carriers for odorant molecules from the external environment to the olfactory neurons. Furthermore, OBPs can bind, solubilize, and transport hydrophobic stimuli to chemoreceptors across the aqueous sensilla lymph. OBPs can also buffer sudden changes in odorant levels and are involved in hygroreception.^[^ ^92^ ^]^ The potential of using OBP to develop biosensors began to be reported nearly two decades ago with preliminary studies on the preparation of Langmuir–Blodgett films containing recombinant rat OBP1 and OBP1F immobilized on the surface of EIS sensors.^[^ ^93^ ^]^OBPs are a class of small molecular water‐soluble proteins that act as carriers of odor molecules from the outside to olfactory neurons.^[^ ^94^ ^]^ OBPs are hydrophilic on the surface and hydrophobic inside. When lipid‐soluble odor molecules enter the nasal mucus or cilia, they can bind to the amino acid residues in the cone of OBPs to interact to form structurally stable complexes. The odorant bound to OBPs binds to ORs with the transport of nasal mucus or cilia. The difference between vertebrate and insect OBPs lies in their 3D structure. Vertebrate OBPs belongs to the lipocalin superfamily and are typical *β*‐barrel structures with calyx‐shaped cavities bound by eight antiparallel *β*‐sheets with a small *α*‐helix at the C‐terminus.^[^ ^95^ ^]^ The calyx‐shaped cavities are nonpolar and therefore able to bind hydrophobic odorants. Insect OBPs are a separate gene family consisting of six *α*‐helical structural domains folded into a very compact globular structure containing six conserved cysteine sites that can form three pairs of disulfide bonds between them to maintain the stability of their protein structure.^[^ ^96^ ^]^ Their selectivity to hydrophobic ligands is not as high as known for ORs, but fine discrimination has been reported in several cases, such as the two enantiomers of carvone bound with different affinities by the pig OBP or the panda OBP5 able to distinguish between stearic, oleic, and linoleic acid,^[^ ^97^ ^]^ as well as isomers of oleic acid differing for the position of the double bond.^[^ ^98^ ^]^ as well as isomers of oleic acid differing in the position of the double bond.^[^ ^98^ ^]^ OBPs are involved in many hydrophobic ligands that exhibit affinity in the micromolar range. The high stability of some of these proteins and their diversity makes them increasingly popular for constructing biological odor sensors (**Figure** **5**a).

Compared to ORs, OBPs have better stability and resistance to degradation by temperature, pH, or protease digestion.^[^ ^99^ ^]^ OBPs can be produced in bacteria with relatively large amounts (20–40 mg L^−1^) and are easily purified by simple column chromatography. These advantages allow OBPs to maintain their functionality and activity despite harsh environments. Gao et al. report the development of a B‐nose based on an AgOBP‐functionalized SiNW (AgOBP‐SiNW) array against a selected group of human‐derived odorants. Working under ambient conditions, the B‐nose device achieved excellent sensitivity down to 2 ppb and highly specific structural selectivity (Figure 5b). The ability of OBPs to serve as recognition elements for biosensors has another important feature: their structures can be modified by simple fixed‐point mutagenesis to alter their affinity and selectivity for ligands and become tailored sensing elements.^[^ ^100^ ^]^ This possibility was further explored in a recent study carried out by Hurot et al. in which, for the first time, three OBP derivatives with different custom binding properties of rat OBP3 were used in combination with the optical transduction system SPR, to develop a highly sensitive and selective analysis of VOCs in solution, all with detection limits in the micromolar range.^[^^]^ In another study, it was also possible to demonstrate that amino acid substitutions in the binding pocket for OBP lead to significant changes in binding affinity.^[^ ^55a^ ^]^ The substitution of residual amino groups at the S82P position of wild AgamOBP1 showed that it exhibited a higher affinity for drugs such as cocaine‐hydrochloride molecules than the wild‐type protein. AgamOBP1‐S82P was immobilized on QCM, which measures mass variations according to changes in the frequency of a quartz crystal resonator, and the biosensor was able to detect cocaine hydrochloride (Figure 5c). This result would not be possible using wild‐type AgamOBP1, highlighting the advantage of modifying the binding properties of OBPs used as sensing elements in biosensors.

The thermal and protein degradation stability, small size, ease of production, and purification of OBP, together with the available structural information allowing the design and synthesis of mutants, make it one of the best candidates for biosensing elements. Examples of the application of OBPs in odor sensing are shown in **Table** **3**. Despite the many structural advantages of OBPs, little research has been done on the use of OBPs as biosensors. Research on OBPs has mainly focused on insects. A review of the literature suggests that vertebrate OBPs have received much less attention than insect OBPs. In particular, they have been studied almost exclusively in mammals. On the other hand, OBPs are odor carriers and are not involved in the detection of odors in general. OBPs have not been studied as extensively as ORs.

**Table 3:** OBP‐based biosensors

Protein	System	VOCs	Immobilization technique	Transducer	Limit of detection	Application	Refs.
pOBP	*E. coli*	Benzene	CNT	EIS	64 pm	Lung cancer, environmental testing	[101]
OBP1, OBP47 (Anopheles gambiae)	*E. coli*	3,4‐Methylenedioxy methamphetamine, cocaine hydrochloride	SAM	QCM	0.01 nm	Drug testing	[55a]
AgOBP5, AgOBP6	*E. coli*	Nonanoic acid, linalool, methyl dodecanoate	CNT	FET	2 ppb	–	[15b]
Pobpf88w	*E. coli*	3‐Isobutyl‐2‐methoxypyrazine, 3‐nitrotoluene, 2‐amino‐4,6‐dinitrotoluene	Nanoparticles	SAW	ppb	Narcotics and explosives detection	[66]
OBP14 (Apis mellifera ligustica)	*E. coli*	Homovanillic acid and related compounds	rGO	FET	4 µm	Agriculture	[102]
OBP3	*E. coli*	Octanal	CNT	FET	0.01 ppm	Food evaluation	[103]
AgamOBP1	*E. coli*	Indole	Nitrocellulose membrane	Lateral flow biosensor	5 ppb	*E. coli* contamination	[104]
HillOBPs	*E. coli*	2‐Methyl butyraldehyde, iso‐valeraldehyde	–	QCM	4 ppm	Pest control	[65]

### 3.3 Synthetic Peptides

In recent years, much attention has been paid to finding biologically‐inspired materials that can mimic the properties of biological receptors. To obtain better stability, synthetic peptides based on ORs and OBPs instead of whole proteins can be accomplished, for instance. Peptides do not require tertiary structures and lipid membranes to remain stable in solution. Peptides are sequences of amino acids of varying lengths and sequence compositions. The chemical structure of amino acids that occur in protein varies only in the R‐group at the carbon in alpha position, C*α*, and are referred to as *α*‐amino acids.^[^ ^105^ ^]^ Peptides adopt a specific conformation based on the position of each R‐group in the amino acid sequence, and the secondary structure of peptides is driven by noncovalent intermolecular interactions such as hydrogen bonding, van der Waals forces, *π*‐stacking, and hydrophobic and electrostatic interactions. *α*‐Helical synthetic peptides in which the parallel and oriented arrangement of hydrogen bonds along the helical axis together produce a strong electric dipole moment that makes peptides susceptible to electric and magnetic fields, which makes them suitable for biosensors.^[^ ^106^ ^]^ On the other hand, the smaller size of peptides makes them easier to be immobilized in an aligned and predefined form on the biosensor surface.^[^ ^107^ ^]^ Peptides have been used in a wide variety of applications, including biosensors coupled to transducers or molecular beacon probes that contribute to signal detection (**Tables** **4**).

**Table 4:** Peptides‐based biosensors

Peptide	VOCs	Transductor	Limit of detection	Applications	Refs.
Cys‐TNT‐BP (virtual screening)	TNT	SPR	0.62 ppb	Environmental monitoring, food quality control, chemical warfare, explosive detection	[106b]
detect dinitrotoluene (DNT)‐bp, DNT‐nbp (virtual screening)	DNT	Resistance analyzer	2.43 ppb	–	[114]
ORPs (OR‐based design)	TMA	DC probes and a resistance analyzer	0.01 ppt	Environmental safety, food quality, and healthcare	[115]
hORp193 (OR‐based design)	Acetic acid	QCM	2 ± 1 ppm	Food safety	[26b]
HarmoBP7‐bp (OBP‐based design)	Long‐chain aliphatic aldehydes	Piezoelectric transducers	14 ppm	Long‐chain aliphatic aldehydes	[116]
LUSH OBP (OBP‐based Design)	3‐Methyl‐1‐butanol	FET	1 fm	Food safety	[108]
GP1/GP2 (Phage display)	Benzene/toluene, xylene, hexane	Microcantilever	121 ppb/2.2 ppm (toluene), 28 ppm (xylene), 1 ppm (hexane)	–	[117]
OR744‐bp (OR‐based design)	1‐Hexanol, 1‐pentanol	QCM	2–3 ppm (1‐hexanol), 3–5 ppm (1‐pentanol)	Salmonella contamination in beef	[118]

The design of peptides with ligand specificity based on the binding regions of OR and OBP is somewhat limited by the diversity of odorants known to bind to these proteins. The production and application of three different peptides from insect OBP in biosensors for detecting 3‐methylbutanol and trinitrotoluene (TNT) have been reported.^[^ ^108^ ^]^ Therefore, different research groups have been trying to design peptides with ligand specificity based on the binding regions of OR and OBP. Recently, approaches such as virtual screening, phage display, and combinatorial peptide libraries have expanded the range of peptide sequences with unique physicochemical properties, improving affinity and selectivity for odorant molecules.^[^ ^109^ ^]^ The previous approach can be limited when there is no natural ligand inventoried for a target molecule. In this context, virtual screening represents an interesting alternative, which allows rapid selection of ligands with high affinity by specific peptides, reflected in the time saved in bench experiments. Four derived peptides from different sequences of hOR1E1 were synthesized using virtual screening and immobilized on a sensor to detect ammonia, acetic acid, and TMA.^[^ ^110^ ^]^ Derivative peptides mimicking the HarmOBP7 binding site were synthesized using a virtual screening method, and biosensors constructed based on OBP‐derived peptides could selectively bind octanal and acetaldehyde. In addition to virtual screening that allows the design and synthesis of synthetic peptides with high ligand affinity, phage display library screening of peptide ligands is an excellent technique to select peptides with high affinity for the target.^[^ ^111^ ^]^ A large number of protein‐binding oligopeptide sequences have been identified by using phage libraries. It should be noted that the results using phage‐displayed peptides and chemically synthesized peptides were slightly different. The binding of the peptide to the ligand is supplemented by the phage‐derived side effects such as the presence of linker amino acid on phage and the increase of structural rigidity by displaying on phage rather than a free peptide. In this study, the peptides binding to benzaldehyde were screened from a phage display library. The peptide sequences showing the highest binding activity were NPAATMA, SIFPVSR, and MPRLPPA. The binding in the gas phase was also confirmed using a candidate peptide‐immobilized ZnO nanowire structure.^[^ ^112^ ^]^ Another approach to designing selective peptide‐based biosensors is using fragments of the antibodies produced from specific cells. One of the examples is the TNT‐binding peptide, being highly specific to the TNT‐designed form complementarity determining region in the anti‐TNT monoclonal antibody.^[^ ^113^ ^]^ In summary, using different methods of preparing peptides broadens the range of peptide sequences with different physicochemical properties and improves the affinity and selectivity of odorants.

The synthetic nature, stability, and high‐yield production of peptides are promising properties. Peptides are alternatives to OR or OBP to construct biosensors. Applying protein fragments such as ligand binding regions or synthetic peptides belongs to a new trend in using biosensors for detecting simple odor compounds. A feature of this application is that the construction of the sensor requires knowledge of the binding pair of ORs and suitable ligands, rather than the complete olfactory signal transmission mechanism. This fact makes peptides one of the most popular biometric components. From the researchers' point of view, it is important to design and screen different structural and functional molecules to better solve challenging biosensor problems and improve the sensing performance.

### 3.4 Molecularly Imprinted Polymer

MIPs are tailor‐made synthetic materials with artificially generated recognition sites able to specifically rebind a target compound in preference to other closely related compounds. In the sensing area, MIPs are synthetic biomimetic recognition element analogs of natural and biological antibody systems.^[^ ^119^ ^]^ They work on a “lock and key” mechanism. MIPs have the potential to have high specificity and selectivity to target molecules with the explicit advantages of environmental durability and low cost. Natural receptors, for example, are generally stored and used at temperatures similar to those of the human body, while MIPs, which are based on a polymer host, can typically be stored indefinitely, do not require specific environmental storage conditions, and can be used over a much wider temperature range. MIPs are considered a promising way to increase selectivity and sensitivity for small molecule detection.^[^ ^37^, ^120^ ^]^

MIPs and their integration with various transducer systems are many of the utmost considerable techniques for biorecognition. A fusion of transducers and MIPs devise a unique and powerful device for biosensing.^[^ ^37^ ^]^ These polymeric biosensors were already studied and demonstrated an encouraging perspective to detect target analytes.^[^ ^121^ ^]^ MIP combined with QCM could improve both the selectivity and sensitivity of gas sensors. A gas sensor with a QCM electrode modified with formaldehyde‐MIPs using polyvinyl chloride as the encapsulation material and tetrahydrofuran as the solvent was reported by Liu et al. This MIP‐based QCM sensor measured the CHO content in fresh shrimp samples with a detection limit of 10.72 ng mL^−1^ and a detection rate in the range of 97.56–98.60%.^[^ ^122^ ^]^ Tang et al. electropolymerized an MIP layer on a TiO_2_ nanotube gas array/Ti sheet. This molecularly imprinted gas sensor detected formaldehyde in the ppm range.^[^ ^123^ ^]^ Recently, Völkle et al. proposed a chemiresistor‐based sensor for the detection of the plant volatile limonene. A polystyrene‐based MIPs and conductive cohybrid were coated on the electrode surface of the QCM and compared to the QCM without MIP. The hybrid MIP produces a strong signal enhancement during the QCM measurements, allowing for the detection of limonene gas concentrations as low as 50 ppm.^[^ ^124^ ^]^MIP can improve the selectivity of localized SPR sensors toward VOCs such as *α*‐pinene, volatile organic acids, and terpenes. For example, a novel localized SPR sensor array based on gold NPs and MISGs (MISG‐localized SPR) has been proposed for the effective recognition of volatile organic acids.^[^ ^125^ ^]^

MIPs have adaptable recognition properties that can be logically tailored. Synthesis of MIPs is easy, straightforward, and inexpensive. MIPs exhibit stability and robustness in both organic and aqueous solvents and their recognition properties did not deteriorate when treated in an autoclave at 120 °C. However, despite its numerous advantages, molecularly imprinted‐based biosensors could suffer considerable limitations in particular domains due to inadequate recognition‐site accessibility, low potential binding sites, and heterogeneous binding.

### 3.5 Olfactory Cells and Olfactory Mucosa Tissue

**Figure 6:**
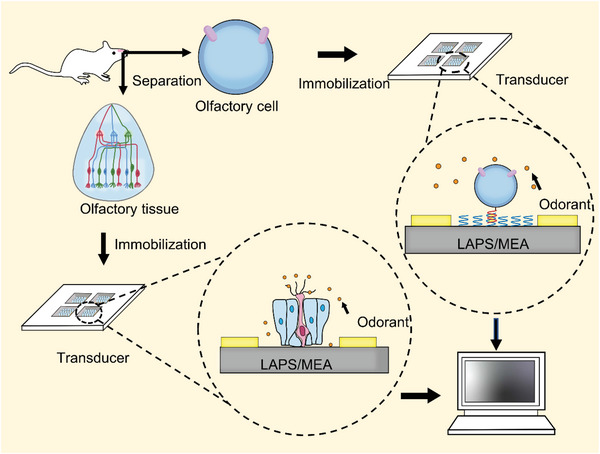
Recording extracellular potentials of olfactory receptor neurons in intact epithelium by LAPS or MEA. Olfactory epithelial and olfactory receptor cells are isolated from rats and immobilized on the surface of MEA or LAPS chips for several days in culture. Olfactory mucosa tissue can be directly coated on the sensor surface, while olfactory cells are immobilized on the sensor surface by double‐stranded DNA or a mixture of poly‐l‐ornithine and laminin.

In addition to the challenges mentioned above, the lifetime of the protein or peptide immobilized on the biosensor is also an important consideration. Proteins immobilized on sensors are commonly at risk of degradation or denaturation, preventing receptor binding to target analytes and impairing their chemosensory function.^[^ ^41^ ^]^ Although peptides are more stable and flexible to reusability than proteins, their lifespan is still limited. Deactivation of biomolecular components makes the sensor ineffective, and the components have to be constantly replaced. An effective way to address these challenges is by using living cells or tissues. Living cells require suitable environmental conditions to maintain viability within the sensor platform, which can readily be cultured for several weeks or months. Furthermore, homeostatic mechanisms within living cells enable cells to adjust to changes in the environment to continue their chemosensory function. One key feature of using olfactory cells and olfactory mucosa tissue as biorecognition elements for sensors is that the signals generated are almost identical to those produced by OSN.^[^ ^126^ ^]^ Various biosensors based on bionic olfactory cells/tissues have been developed (**Figure** **6**).

Typically, olfactory cells are cultured directly on the sensor surface, which results in a random distribution of cells and uncontrollable coupling to the sensor. This obvious drawback leads to various limitations in the performance and applications of cell‐based biosensors, such as poor stability and reproducibility (**Table** **5**). To improve the biosensor performance, the sensor surface can be coated with a mixture of poly‐l‐ornithine and laminin before olfactory cell immobilization. Recently, the DNA‐directed cell immobilization method may provide new solutions to this problem, which can generate desirable cell distribution controlled by the pattern of single‐stranded DNA (ssDNA) probes on the solid surface.^[^ ^127^ ^]^ ssDNA is covalently linked to the cytoplasmic membrane of the cell, and the complementary ssDNA is immobilized on the sensor surface as a probe. This DNA‐directed fixation method has been used to achieve a controlled and efficient coupling between the olfactory cells of rats and MEA chips.^[^ ^128^ ^]^ This approach has been widely applied in complex cell pattern construction,^[^ ^129^ ^]^ capturing living cells for cellular analysis, and nanoliposomes.

**Table 5:** Advantages and disadvantages associated with commonly used biomaterials in biosensors development

Biosensing material	Advantages	Disadvantages	Refs.
ORs	Can be acquired from a large variety of expression systems: *S. cerevisiae*, *E. coli*, and cell‐free. They generate changes in electrical properties within themselves upon odorant binding. Possibility for large scale production. Compared to olfactory cells and olfactory epithelium, they are easier to store for long periods and easier to use in practice, which contributes to the miniaturization and convenience of biosensors. Genetic engineering can be used to be able to add tags or other specific sequences to facilitate their purification and immobilization on the sensor. Very high sensitivity while using the whole protein. Can identify small variations in odorants based on their structural construction and concentration. Possibility of biomimetic approach and virtual design.	OR production is time‐consuming, labor‐intensive, and relatively inefficient. Complicated structure of ORs. Easily lose their functions by many external factors, such as heat and physical forces, which can affect the structure of OR proteins. Hard to immobilize onto the secondary transducer.	[131]
OBPs	Extremely stable to temperature, organic solvents, proteolytic digestion, pH, and proteases. Easier separation and purification than OR production methods. Can be tailored through mutagenesis.	Identifying olfactory OBP specific to an odorant may be time‐consuming, labor‐intensive, and expensive. High cost of production, difficult to manufacture, and high reproducibility of proteins.	[132]
Synthetic peptides	Excellent stability and reproducibility, Smaller size of the peptide makes it easier to fix it in an aligned and predetermined form on the surface of the secondary transducer. Possibility to synthesize different peptide sequence with Fmoc method. Can be stored for long periods of time, Keep intrinsic property of the ORs themselves. Can be easily modified in specific sites.	Synthesis limited to specific number of amino acids. Expensive Fmoc reagents and high volumes of toxic solvents and reagents during synthesis.	[133]
Olfactory cells	Generate signals which can be similar to those produced by ORNs. May facilitate the mechanism of OR sensing principles. Suitable for physical absorption. Can detect real‐time extracellular signals under odorant stimulations for prolonged periods. Very high sensitivity while using the whole protein. Provide insight into the physiological effect between odorant molecules and olfactory cells.	Hard to manufacture and store. Isolation and in vitro culturing of OSN cells is difficult, so their practical application is limited. Relatively high cost. Limited applicability to some secondary transducers. In mammalian systems, the ability to readily transfect reporter genes limits the cell types to those that are tumor derived. The potential can be measured only at a limited number of sites, such as the tip of an individual microelectrode.	[134]
Olfactory mucosa tissue	The functional receptor unit of cilia on each olfactory receptor cell would not be damaged. The intact epithelium allowed simpler acute preparation and easier visualization, without strictly controlled cell culture conditions (*i.e*., nutrient media, pH, temperature, and sterile environment). Extracellular compartments present in vivo (including supporting cells and basal cells) were preserved. Preserves natural state of the neuronal populations and can be obtained easily, The mucus layer with odorant binding protein generated by Bowman's glands and supporting cells were preserved. Signal of many cells can be detected synchronously.	Need to kill animals. Should be kept in standard perfusate with suitable temperature, humidity, and nutrient solution to maintain native state and their biological activities. Hard to manufacture with high repeatability. Significantly reduced signal strength and quality in long‐term usage.	[135]

Isolated neuronal cells can adapt to the extracellular environment and perform odor information transmission, but it is challenging to maintain their natural state. Using olfactory epithelial tissue as the sensitive element allows signal production and has high sensitivity and fast response. A study coupling olfactory epithelial tissue of adult rats to MEA was able to record different oscillatory signal patterns for different odorants.^[^ ^130^ ^]^ Another report coupled rat olfactory epithelial tissue with LAPS and was able to monitor the benefits of region‐specific activity within the olfactory epithelial tissue. The use of tissue sections as sensitive elements has not been standardized, ORs are not uniformly distributed throughout the olfactory epithelial tissue, and they are expressed in different regions. The ability of ORs to recognize specific chemicals may be compromised during the isolation of olfactory epithelial tissues.

Olfactory cell and tissue‐based biosensors use living cells as sensing elements to obtain complete information on olfactory transmission mechanisms. Cells and tissues can be extracted from olfaction and cultured in vitro. Then, electrical activities directly related to cellular functions can be detected by microelectronic sensor chips. Applications of olfactory cell‐ and tissue‐based biosensors are in many fields ranging from the environment to biomedical diagnostics. Regarding potential implementation, approaches to applying olfactory cells and tissues as biometric components encounter major obstacles: the lack of analytical methods characterized by high sensitivity, specificity, reproducibility, and reliability.

## 4 Applications of Bioelectronic Nose

**Figure 7:**
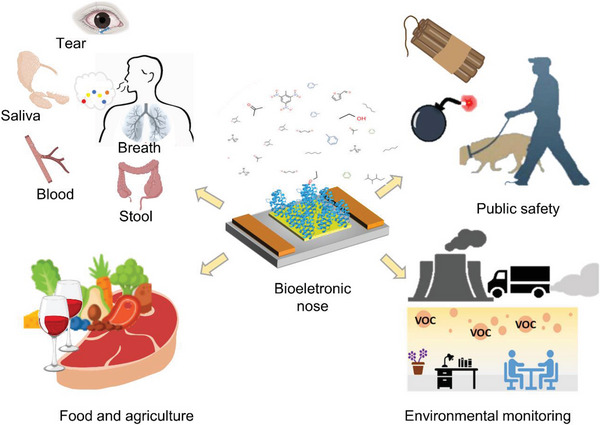
Applications of B‐EN in the areas of biomedicine, food and agriculture, public safety, and environmental monitoring.

Various kinds of B‐ENs are being applied for olfactory analysis owing to the unique electrical and biological properties of biosensors with biological recognition elements, thereby elevating the sensitivity and specificity of detection. After years of development, B‐EN has been used in a wide range of biomedical applications, as well as food safety, public safety, and environmental testing. Four main application areas are included, as shown in **Figure** **7**.

### 4.1 Applications in Medical Diagnosis

A large number of deaths due to multifactorial diseases (cancer, respiratory diseases, cardiovascular diseases, etc.) is mainly due to late diagnosis, which limits treatment and increases healthcare costs. As a result of normal metabolic function, the healthy human body produces large amounts of VOCs, many of which are derived from commensal microorganisms and internal organ systems within the body. Due to different metabolism, different pathogens produce characteristic VOC profiles. The accurate identification of VOCs emitted from the body can indeed provide information on health and metabolic pathological conditions.^[^ ^136^ ^]^ In particular, VOC sensors have gained considerable interest for the selective and continuous diagnosis of various physiological and pathological states acting as biomarkers to identify numerous diseases in a noninvasive way. B‐EN using natural receptors potentially hold promise in biomedical applications, including clinical diagnosis.^[^ ^137^ ^]^

Hexanal is considered to be a biomarker for lung cancer. Janfaza et al. developed an MIP‐based B‐EN device for the detection of hexanal.^[^ ^138^ ^]^ Molecularly imprinted nanoparticles and multiwalled nanotubes were used in the sensor to form the sensor capable of selectively detecting gaseous hexanal at room temperature. It works in the 10 to 200 ppm concentration range and has a detection limit of 10 ppm. Octenol is also a human metabolite for cancer and Coulomb's disease. Octenol can cause neurodegeneration and cytotoxicity. Yamada et al. proposed a VOC sensor using insect olfactory receptors reconstituted as lipid bilayers.^[^ ^87^ ^]^ The lipid bilayer is integrated on a chip with a gas flow system and the VOC is delivered to OR‐Orco through the gas flow system to achieve high detection sensitivity. The sensor is capable of detecting octenol in human breath with a sensitivity of 0.5 ppb. TMA is also considered highly hazardous and can cause serious health problems and odor nuisance. A high level of TMA in urine can be a symptom of a metabolic disorder called trimethylaminuria. A method to fabricate a high‐performance TMA sensor by chemically conjugating olfactory receptor‐derived peptides (ORPs) to SWCNTs on interdigital electrodes was recently presented by Wang et al.^[^ ^115^ ^]^ Novel carbon nanotube (CNT)‐FETs are functionalized with 12‐mer peptides identified using phage display.^[^ ^139^ ^]^ Peptide‐CNT FETs discriminated of four different breath‐related VOCs (isopropyl alcohol, acetone, isoprene, and toluene) suggesting that CNT‐FETs have the potential for wearable breath monitoring applications such as personal health diagnosis and real‐time human performance assessments.

Portable electronic technologies are an important part of the overall healthcare system, with an enormous capacity for surveillance, treatment, diagnosis, fitness, and well‐being. Together, they will improve preventive actions and better view their well‐being, combined with treatment instruments found in hospitals and emergency care facilities. Continued advances in technology and increased use of biosensors in diverse applications are the drivers of market development. Wearable biosensors have enhanced life quality. The data generated by wearable B‐EN can be transmitted to smartphones via NFC, WiFi, and Bluetooth technologies, enabling users to be aware of their surroundings or body conditions in real‐time. With the rise of on‐chip integrated systems, battery‐free devices, and advanced manufacturing materials, the overall wearable B‐EN size will become smaller, and biosensing stability and uptime will be enhanced. Wearable B‐ENs have great commercial potential in medical diagnostics.

### 4.2 Applications in Food Safety

Food safety is an important critical issue for the modern food industry. Contaminants, bacteria, and toxins, can enter the food during production and storage or be produced in the food by reacting with compounds.^[^ ^140^ ^]^ Foods with high levels of protein are among the most perishable foods, in which the decarboxylation and deamination reactions are caused by amino acid degradation under the action of endogenous and microbial enzymes, leading to the formation of toxic biogenic and volatile amines. The off‐flavors produced by volatile amines reduce the organoleptic quality of foods, and biogenic amines are usually toxic and harmful to humans when consumed. Therefore, monitoring volatile and biogenic amine concentrations in food is a reliable criterion for food freshness, quality, and safety.

A wireless portable B‐EN device was reported to detect multiplex monitoring food freshness or spoilage.^[^ ^26d^ ^]^ In this study, BE‐nose integrated with trace amine‐associated receptor‐NDs, allowing food quality monitoring via the detection of food spoilage indicators, including the biogenic amines cadaverine (CV) and putrescine (PT). In the gas sensor system, the detection limits were 26.48 ppb for CV and 7.29 ppb for PT. Wang et al. reported an ORPs‐based B‐EN for seafood quality assessment.^[^ ^115^ ^]^ The ORP was synthesized and directly immobilized onto SWCNTs. The ORPs were connected to the SWCNTs by Steghlich esterification reaction and natural chemistry, and different concentrations of vaporized TMA and real food TMA were measured. The results show that the fabricated ORP sensor enables the detection of gaseous TMA at concentrations as low as 0.01 ppt and high‐performance detection of TMA generated by different types of spoiled foods. B‐EN allows early assessment of food contamination due to their simple operating procedures and rapid detection of gases. B‐EN may be a promising tool for food quality and safety testing.

### 4.3 Applications in Public Safety

In recent decades, various forms of terrorist incidents have occurred frequently, and public safety has been the focus of international attention. To ensure the safety of citizens, governments have stepped up their efforts to inspect various dangerous goods, especially explosives and drugs. The dog's nose sensitivity can reach 10 million times that of humans, and can easily identify more than 2 million different odors. In airports, customs, and other places, trained dogs can search for explosives and drugs among many odors. However, there are some limitations to this. For example, the training and maintenance costs of police dogs are high. In addition, the olfactory nerves of dogs are stimulated by the same odor repeatedly, which tends to produce olfactory fatigue. As a result, police dogs are unable to search for drugs and explosives continuously.^[^ ^141^ ^]^ Therefore, an alternative method is needed to replace dogs, and B‐ENs are a good choice.

With the development of modern analytical techniques, more and more B‐ENs for the detection of various explosives have been developed. Komikawa et al. developed a new 3D peptide‐based “peptide matrix” structure to improve the affinity of TNT‐binding peptide probes. The unique structure of the peptide matrix is rigidly constructed by multiple TNT‐binding peptide fragments and is assembled on an SPR sensor chip, and this peptide matrix greatly improved the ability to capture a TNT.^[^ ^142^ ^]^ Lee et al. designed and fabricated a highly sensitive and selective detect dinitrotoluene (DNT) gas sensor using DNT‐specific binding peptide‐functionalized rGO. The results show that the sensor has high sensitivity with a detection limit of ≈2.43 ppb and provides reproducible and regenerative surfaces for use in practical field applications.^[^ ^143^ ^]^ Genetic engineering of a bacteriophage is a promising way to develop a highly functional biosensor. Optical B‐EN of outstanding sensitivity and selectivity toward VOCs implemented with genetically engineered bacteriophage was recently presented by Park et al. They demonstrated a fast optical B‐EN with high selectivity for gaseous explosives such as TNT, DNT, cyclotrimethylenetrinitramine.^[^ ^144^ ^]^ Scorsone selected a series of natural and modified ligand‐binding proteins belonging to the OBPs and major urinary proteins families that were integrated into a nanodiamond‐coated SAW sensor. A sensor array was created based on the ligand‐binding affinity of these compounds to protein‐binding pockets, and this sensor can detect not only explosives, but also drugs such as cannabidiol, cocaine, and heroin.^[^ ^145^ ^]^ In summary, the ease of operation, rapid analysis, and low error rate make B‐ENs a potential replacement for dogs and other detection methods in detecting and analyzing explosives.

### 4.4 Applications in Environmental Monitoring

Detection and regulation of harmful chemical emissions in the atmosphere have become a major concern for many countries across the globe. Tons of organic and inorganic contaminants are released into the air, soil, and water, which can pose many health threats not only to humans but also to plants and animals.^[^ ^4a^ ^]^ In these situations, B‐EN is the best solution. B‐ENs are based on various operating principles and can be used to control chemical contamination in many environmental applications.^[^ ^47^, ^146^ ^]^ They can be widely used for environmental monitoring of urban pollutant emissions for air pollution and water pollution monitoring purposes.^[^ ^147^ ^]^ B‐EN can quickly detect leaks of toxic or hazardous substances in ducts or industrial plants and can potentially warn of the accumulation of organic solvents or explosive fumes.

Geosmin (GSM) and 2‐methylisoborneol (MIB), mainly produced by bacteria, are representative odor compounds and are indicators of contamination in water supply systems. B‐EN for water quality implementation assessment was constructed using hOR and SWCNT‐FET by Son et al.^[^ ^44e^ ^]^ The B‐EN was able to selectively detect GSM and MIB at concentrations as low as 10 ng L^−1^. Odor discrimination using a cell‐based B‐EN and fluorescent image processing by Sukekawa et al.^[^ ^148^ ^]^ Cells expressing OR13a and OR56a were used directly as sensors, and GSM and 1‐octen‐3‐ol were used as target odorants. The results suggest that the combination of B‐EN and image processing techniques has the potential to discriminate between different odorant molecules, even if the cells are placed at random. NO_2_ is a major air pollutant, and its exposure can have adverse short‐term (inflammation in the respiratory tract) and long‐term (lung infections and respiratory failure) effects on humans. An artificial olfactory system based on a spiking neural network (SNN) and FET‐type gas sensors for fast and reliable detection of NO_2_ ware proposed by Kwon et al.^[^ ^149^ ^]^ The FET‐type gas sensors with an In_2_O_3_ film were fabricated to detect NO_2_ gas. It was shown that the proposed SNN has significant immunity to the inevitable read fluctuation of the gas sensor. Consequently, the proposed artificial olfactory system based on the hardware‐based SNN and the FET‐type gas sensors shows a highly reliable performance in fast toxic gas detection with low power consumption.

## 5 Conclusions and Future Outlook

A variety of artificial olfactory systems and sensing techniques with high selectivity and sensitivity have been developed by mimicking biological olfaction systems. With an increasing understanding of ORs and OBPs, synthetic proteins and peptides are increasingly being used as substitutes for tissues and cells to recognize specific odorants. Artificial olfactory biohybrid systems have strong potential in food safety, environmental detection, medical analysis, and national defense.

Despite its promising prospects, in practice, the OR‐based biosensor is an early‐stage technology, and so far, no commercialized B‐EN has been marketed. Many fundamental challenges and obstacles related to achieving highly stable and reliable continuous biomaterials need to be settled. Only a few cognate ligands exist in ORs currently available odorant‐binding repertoire. Therefore, to produce the desired OR, various OR genes have to be cloned and expressed. The immobilization of ORs on the sensor surface remains an important factor in the field of biosensor development. Since OR proteins have to remain in a lipophilic environment to retain their structure and function, the procedures used to immobilize OR proteins or cells expressing OR on solid surfaces have to be conducted under mild ambient conditions such as at the proper pH, temperature, etc. On the other hand, the development of smart biosensor systems still faces many challenges. From the perspective of materials, to combine structural functional materials and sensing functional materials well, they not only have excellent combination characteristics but also have similar preparation and synthesis processes. The repeatability and stability of the sensor array are easily influenced by environmental factors (such as humidity, temperature, and vibration), which lead to the instability of the measurement data. For gases or solid particles that are difficult to be adsorbed by common substrates, the long‐term stability of the sensor in the environment (that is, the signal is not shielded by interfering substances) and the sensitivity of the signal acquisition and transportation system needs to be improved.

In the context of the biological transformation, odorant detecting biosensors assume a special position as they are not only considered an application of a biotechnology interface (BTI) based system, but can also be seen as enablers of superordinate BTI‐based systems (e.g., deployed in a bioreactor). However, for odorant‐detecting biosensors to make the step from biointegrated to truly biointelligent systems, further development in the area of pattern recognition (multisensing capability) is still necessary. Due to the use of ORs as one of the measurement elements, it mimics the operating principle of the human sense of smell in the most precise way. Therefore, B‐EN will be a potent tool for smell visualization, but only if two technologies are completed. First, a multichannel array‐sensing system has to be applied to integrate all of the ORs into a single chip to mimic the performance of human nose. Second, the processing technique of the multichannel system signals should be simultaneously established with the conversion of the signals to visual images.

With the rise and development of technologies such as chips, internet, 3D/4D printing, advanced materials, artificial intelligence, and other cutting‐edge approaches, it provides a robust evolution drive to realize smart sensing, odor digitization, olfactory virtual reality, and intelligent odor intervention. In such a context, highly precise and fast odor sensing could be realized for environment detection, intelligent robot development, earthquake sniff rescue, and customs security check. Additionally, it also could open up new possibilities not only for the service industries, such as advertisements with flavors, immersive virtual reality video games with flavors, avoiding fatigued car driving through odor intervention, assisting the deafblind through scent, and achieving emotional stress relaxation through comfortable scents, but also for medical industries, such as drug screening, sniffing out diseases by human body odor, and recovering memories of people with mental illness through familiar flavors.

## Conflict of Interest

The authors declare no conflict of interest.
